# Local Ecological Knowledge and Scientific Data Reveal Overexploitation by Multigear Artisanal Fisheries in the Southwestern Atlantic

**DOI:** 10.1371/journal.pone.0110332

**Published:** 2014-10-15

**Authors:** Mariana G. Bender, Gustavo R. Machado, Paulo José de Azevedo Silva, Sergio R. Floeter, Cassiano Monteiro-Netto, Osmar J. Luiz, Carlos E. L. Ferreira

**Affiliations:** 1 Departamento de Ecologia e Zoologia, Universidade Federal de Santa Catarina, Florianópolis, SC, Brazil; 2 Departamento de Biologia Marinha, Universidade Federal Fluminense, Niteroi, RJ, Brazil; 3 Fundação Instituto de Pesca, Arraial do Cabo, RJ, Brazil; 4 Department of Biological Sciences, Macquarie University, Sydney, NSW, Australia; Aristotle University of Thessaloniki, Greece

## Abstract

In the last decades, a number of studies based on historical records revealed the diversity loss in the oceans and human-induced changes to marine ecosystems. These studies have improved our understanding of the human impacts in the oceans. They also drew attention to the shifting baseline syndrome and the importance of assessing appropriate sources of data in order to build the most reliable environmental baseline. Here we amassed information from artisanal fishermen's local ecological knowledge, fisheries landing data and underwater visual census to assess the decline of fish species in Southeastern Brazil. Interviews with 214 fishermen from line, beach seine and spearfishing revealed a sharp decline in abundance of the bluefish *Pomatomus saltatrix*, the groupers *Epinephelus marginatus, Mycteroperca acutirostris*, *M. bonaci* and *M. microlepis*, and large parrotfishes in the past six decades. Fisheries landing data from a 16-year period support the decline of bluefish as pointed by fishermen's local knowledge, while underwater visual census campaigns show reductions in groupers' abundance and a sharp population decline of the Brazilian endemic parrotfish *Scarus trispinosus*. Despite the marked decline of these fisheries, younger and less experienced fishermen recognized fewer species as overexploited and fishing sites as depleted than older and more experienced fishermen, indicating the occurrence of the shifting baseline syndrome. Here we show both the decline of multigear fisheries catches – combining anecdotal and scientific data – as well as changes in environmental perceptions over generations of fishermen. Managing ocean resources requires looking into the past, and into traditional knowledge, bringing historical baselines to the present and improving public awareness.

## Introduction

Fishing is the most ancient form of exploitation of coastal resources, preceding all other human disturbances to marine ecosystems, such as pollution, eutrophication, habitat loss, disease outbreaks, human induced climate change and species invasions [1]–[4]. Fishing has caused the worldwide depletion of large predatory fishes, including shark populations [5], [6], and driven several species across different ecosystems to ecological extinction [1]. Moreover, fishing is reported to impact fish populations since prehistoric times [2], [7]–[10], affecting species diversity and size [11], [12]. Today, fishing remains as the major source of impact upon marine and coastal environments, contributing to global biodiversity loss [13]–[15]. The continuity of unsustainable historical fishing has left few truly pristine ecosystems in the marine realm [1], [5]. In the Southwestern Atlantic, patterns of marine resources' overexploitation are similar to what have been reported worldwide [5], [14], [16], with widespread population declines and collapsed stocks [17]–[19]. It has been estimated that 23% of all Brazilian marine fish stocks are fully exploited and 33% are overexploited, including species from low trophic levels [20]–[22]. Overfishing has been changing the density and the size structure of reef fish top predators occurring in Brazil [17], [23], consequently elevating their threatened status [24], [25].

Environmental changes and lack of baselines for pristine marine ecosystems have profound implications in our perceptions of what is a natural environment [4], [26]. The compromised ability of people in perceiving environmental modifications and past ecological conditions is called the shifting baseline syndrome (SBS) [27]. This phenomenon was first noted among fisheries scientists, who perceived as a ‘natural’ baseline for stock size and composition the condition they observed at the start of their careers, while failing to incorporate past and historical data [27]–[29]. As generations change, environmental baselines become increasingly shifted, misinforming fisheries management. In order to adjust shifted environmental perceptions, old travel diaries, naturalists' observations, historical data and fishermen' anecdotes are important sources of information [28]–[32], as well archaeological and paleontological data [1], [4]. Baselines constructed from historical data are critical to better gauge and interpret long-term changes and to set appropriate targets for management and restoration [1], [4], [33].

**Figure 1:**
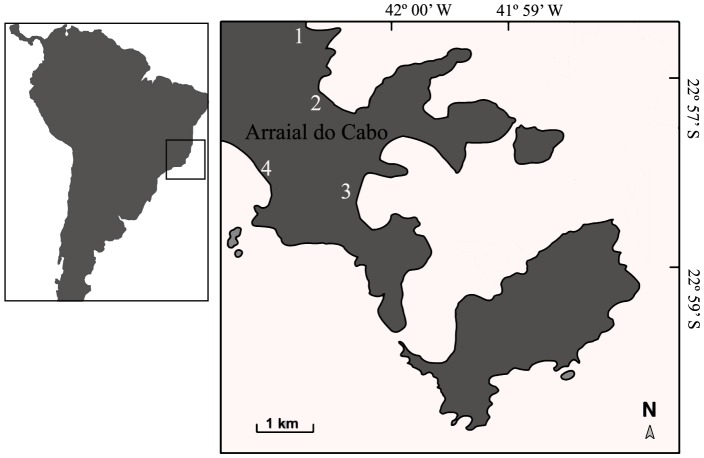
Locations of interview sites at Arraial do Cabo.

The Local Ecological Knowledge (LEK) held by traditional fishing communities is considered an important tool for the assessment and management of tropical fisheries [29], [30], [37]. Local Ecological Knowledge is a set of perceptions and experiences of traditional communities regarding its surrounding natural environment [34], [35], this knowledge being handed-down through generations by cultural transmission [36]. This knowledge includes perceptions of fishing resources – fisheries' composition and abundance patterns, as well as fish species biological and ecological aspects [38], [39] – all of which are important to conservation and management strategies [40]. Because humans have modified natural systems, local perceptions of the status of species and ecosystem resources are unlikely to remain constant over time [41]. Thus, the SBS could influence the validity of LEK derived data, whose use for species and ecosystems assessments is becoming more common [42], [43]. However, specific conditions are required for SBS to occur, which include a combination of environmental events and observer perceptions [44]. Here we combine multiple sources of information to understand the status of local fisheries of Arraial do Cabo, Rio de Janeiro state, Southeastern Brazil (Fig. 1). Specifically, we aim to (i) investigate the occurrence of SBS among fishermen communities of Arraial do Cabo; (ii) utilize LEK to obtain past estimates of abundance and population trends for exploited fish species; and (iii) demonstrate the importance of anecdotal data to adjust perceptions regarding marine resources in the subtropical coast of Brazil.

## Methods

### Study site

Arraial do Cabo (Rio de Janeiro state, Brazil) constitutes a traditional fishing village with approximately 20,000 residents and 1,340 active fishermen. The area was declared a Marine Extractive Reserve (MER) in 1997 – the first of its kind in Brazil – a collaboratively managed marine protected area where only local fishermen are allowed to exploit resources. In Arraial do Cabo, while there are some fishing regulations, enforcement is poor or non-existent, resulting in a typical overexploitation scenario [17]. Among fishing gears, hook and line, gillnet, beach seine and spearfishing are the most used. The beach seine fishery is the most traditional practice, using large canoes and seine nets that encircle passing schools of bluefish (*Pomatomus saltatrix*), mullets (Mugilidae), jacks and trevallies (Carangidae), and tunas (Scombridae). Spearfishing commenced *c.* 50 years ago in Brazil, being today widespread along the coast. The region is formed by an isthmus and two islands dominated by rocky shores and sand beaches. Coastal morphology associated to prevailing winds trigger small-scale upwelling events, favoring a rich marine environment (Fig. 1).

### Data collection

#### Ethical considerations

The Marine Biology Graduate Program at Universidade Federal Fluminense (PPGBM-UFF), Niterói, Rio de Janeiro state, Brazil, approved the data collection of our study that comprised interviews with local fishermen communities. Prior to each interview, fishermen were informed on research purpose and we obtained verbal consent from participants. This methodology was chosen because some interviewees would lack reading and writing skills. Once the participant agreed on being interviewed, we recorded participant consent by writing the participant's name and interview date on questionnaire sheets. This procedure was considered adequate by the Graduate Program review board. We also informed participants that all information provided in interviews would be anonymized.

#### Interviews

To assess fishermen perceptions regarding the status of overexploited fish species, we conducted individual interviews with 214 fishermen – from August 2007 to August 2008 – in Arraial do Cabo, Southeastern Brazilian coast (Fig.1). Interviews with fishermen included three different gears: hook and line, beach seine and spearfishing. As line and beach seine are traditional fishing gears in the region and have been practiced for centuries, this enabled a large number of experienced fishermen to be surveyed. Making possible the unveiling of a rich ecological knowledge related to these gears and a robust historical baseline for local fisheries. Respondents were categorized into three groups according to fishing experience. For line fishing and beach seine fishing the categories were: beginners (<16 years of practice, n = 45), intermediate (16–30 years of practice, n = 50) and experienced (>30 years of practice, n = 43). However, since spearfishing is a recent fishing practice when compared to beach seine and line fishing, experience categories for spearfishermen were adapted to a smaller period: beginners (<8 years of practice, n = 25), intermediate (8–15 years of practice, n = 28) and experienced (>15 years of practice, n = 23).

The survey started a few weeks following arrival to the fishing village. Fishermen were randomly approached at the beach – as they were encountered either going fishing or making repairs on nets and fishing gears – and inquired on the availability to participate on the research. Following this approach, fishermen in different groups (experience category per fishing gear) were selected through snowball sampling [37], [45], where one or more peers indicate the respondent. This method was applied so that we could achieve the target sample size in each experience category and fishing gear (line, beach seine and spearfishing). This method also led us to interview some elder and retired informants at their homes. Respondents were always interviewed separately in order to avoid interfering from others. Our questionnaire constitued of structured and open-ended questions [29] (see File S1 for questionnaire).

The bluefish, *Pomatomus saltatrix* was chosen among various species based on its importance as a resource in the region [45], [47]. We asked fishermen about the best bluefish catch they ever remembered landing and the year in which this catch was made [47]. For the spearfishing practice interviews, we selected four Epinephelidae (groupers) species [48] that are important targets in the region (*Mycteroperca acutirostris*, *M. bonaci*, *M. microlepis* and *Epinephelus marginatus*) [17]. To explore the possible fishing impacts at lower trophic levels, we included in questionnaires four endemic large herbivorous-detritivorous species of the subfamily Scarinae (parrotfishes), namely, *Scarus trispinosus, Sparisoma axillare, Sparisoma frondosum* and *Sparisoma amplum*. These fishes became targets for spearfishing in the last 20 years [17], [20]. For each species we asked divers about the best catch they remembered spearing (number of fishes/individuals), the largest individual they ever caught (in kilograms), and the year in which these catches were made [29]. When fishermen were asked about the largest fishes they had ever caught, the majority of respondents provided information relative to species weight (in kilograms). However, when fishermen referred to fish body length, species length–weight relationships were used to convert to weight [49]. Finally, we assessed shifts in environmental perceptions among generations of fishermen. We inquired each respondent to cite the number of overexploited species and sites in the region, as well as the number of species that were formerly discarded but are now fishing targets. Given the richness of common names for Brazilian reef fish [46], we used photographs to clarify species identifications during the interviews [37].

#### Bluefish landing data

We have included fisheries landing data in our analysis in order to improve the overall understanding of the status of fish stocks in the region. Bluefish (*Pomatomus saltatrix*) catch data (total catch – kg and effort – fishing hours) was recorded daily and pooled over the year during 16 years (1992 to 2008) at the local landing point (Marina dos Pescadores, Porto do Forno) in Arraial do Cabo.

#### Reef fish abundance

The abundance of groupers (Epinephelidae) and parrotfishes (Labridae, Scarinae) was assessed through an underwater visual census (UVC) program performed in Arraial do Cabo region across different years (1992, 2012). The program is led by one of the authors (C.E.L.F.), and includes UVC and other sampling techniques applied to studies/monitoring of the reef community at Arraial do Cabo. Fish density was estimated using 20×2 m strip transects [50]. In each transect, all fishes within the sample area were identified, counted and categorized into five size classes. Data across all sampled sites were pooled together to show temporal changes for target species in Arraial do Cabo region.

### Data analysis

#### Interviews

From the data obtained through interviews with local fishermen we measured the decline in fish species catches, as well as the differences in the set of environmental perceptions across fishermen generations. With the purpose of measuring the decline in fish species abundance and body-size patterns across the years, we applied regressions and plotted (i) the best day catch reported by each respondent, versus the year in which the catch was made; and (ii) the larger individual ever caught by the respondent (kg) versus the year in which a given species was caught. We tested distinct regression types (exponential, polynomial and linear regressions) and *r* ^2^ values were compared to assess the quality of the regression fit to data distribution. Fish catches measured for time intervals are presented in the results section as mean ± standard deviation.

To investigate the potential differences in respondents perceptions, we tested differences in the mean number of overexploited species, overexploited sites, and the mean number of target species (formerly discarded) mentioned by fishermen in different categories (beginner, intermediate, experienced) through one-way ANOVA (α = 0.05; Student-Newman-Keuls post-hoc test) [51]. All data was tested for normality (Kolmogorov-Smirnov test) and transformed when necessary (square root or log transformed) before conducting analysis.

#### Bluefish landing data

In order to measure *P. saltatrix* catch trends across the years (1992 to 2008) from landing data we have applied regression analysis, plotting the catch per unit effort (tons/hour) throughout the years. Again, we tested for distinct regression types (exponential, polynomial and linear regressions) and *r* ^2^ values were compared to assess the quality of the regression fit to data distribution.

#### Reef fish abundance

In order to examine the population decline trends within targeted reef fish families (groupers and parrotfishes) in Arraial do Cabo we tested the Spearman's rank correlation between the mean density (of groupers and parrotfishes) per transect and year of sampling. Only the most abundant and large sized parrotfish species (*Scarus trispinosus, Sparisoma axillare, S. frondosum, S.amplum*) were used for analysis [52].

## Results

### Multiple sources reveal fish species declines

**Figure 2:**
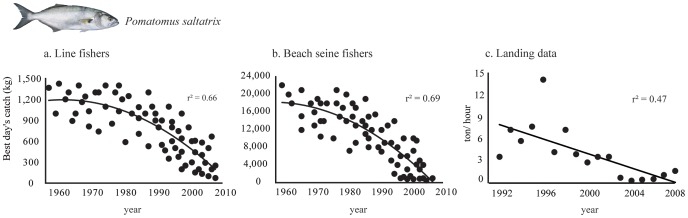
Bluefish *(Pomatomus saltatrix)* catches according to line fishermen (a) and beach seine fishermen (b), both are second order polynomial regressions; and catch per unit effort (t×h^−1^) (c) from 1992 to 2008 in Arraial do Cabo, Brazil.

The pattern for bluefish catches across the years reveals significant reductions in *P. saltatrix* stocks in Arraial do Cabo region over time (Fig. 2a). Captures (mean ±standard deviation) of 1,200 kg (±186) were considered a good catch during the 1960s among line fishermen. This number decreased to an average of 970 kg (±260) in the 1980s. Today, 370 kg (±220) of bluefish is considered a great catch (Fig. 2a). Results reveal that in fifty years (1960–2010) the amount of bluefish caught on a good day catch was reduced by approximately 70%.

Experienced beach seine fishermen also reported great bluefish catches in Arraial do Cabo decades ago, and approximately 80% decline in the past six decades, a pattern similar to that identified by line fishermen (Fig. 2b). During the 1960s, large catches were in the order of 18,000 kg (±3,517) on average. In the 1980s, this number has fallen to13,570 kg (±3,753), and today *P. saltatrix* catches greater than 3,700 kg (±3,340) are rare. Moreover, landing data from *P. saltatrix* catches in the region reveal the same pattern found by accessing the knowledge of local fishermen (Fig. 2c), with a declining trend in bluefish fisheries along 16 years (1992–2008). CPUE varied between 14.2 tons/hour in 1996 and 0.3 tons/hour in 2004, with an annual average of 4 tons hour (Fig. 2c).

**Figure 3:**
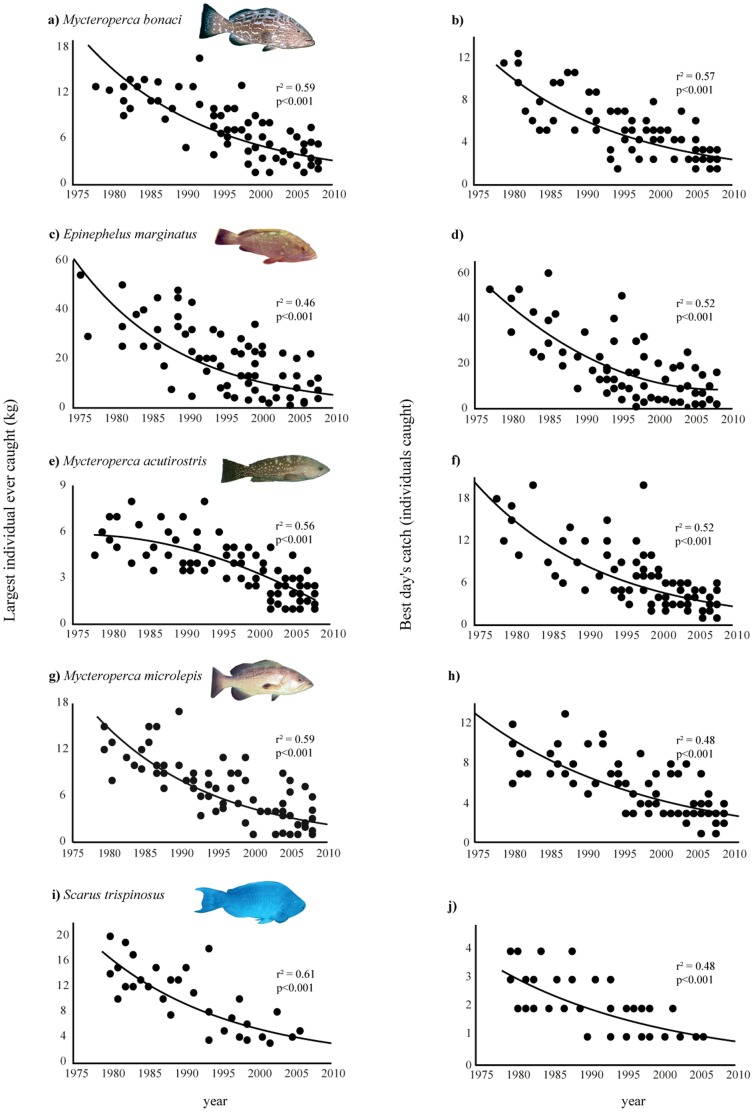
Largest individual (in kilograms) and greatest catch (number of individuals caught) that spearfishermen remembered landing. a) and b) *Mycteroperca bonaci*, second order polynomial regression; c) and d) *Epinephelus marginatus,* exponential regression; e) and f) *Mycteroperca acutirostris*, second order polynomial regression; g) and h) *Mycteroperca microlepis*, second order polynomial regression; i) and j) *Scarus trispinosus*, exponential regression.

Fish species exploited by spearfishing also exhibit declining trends. There was a decrease in the abundance of the assessed Epinephelidae species (Fig. 3). The relationships considering the largest catch and year again indicate that large individuals of *E. marginatus*, *M. bonaci*, *M. acutirostris* and *M. microlepis* have declined in the region, and that the big ones were frequently caught not so far back, in the 1970s (Fig. 3). For the endemic parrotfishes *Sparisoma axillare, S. frondosum* and *S. amplum* there were no significant differences neither in the abundance nor in the size of individuals caught along the years (Fig. S1 in File S1). However, for *Scarus trispinosus*, we have identified reductions on the greatest individuals captured (kg) and on the best catches made (Fig. 3).

**Figure 4:**
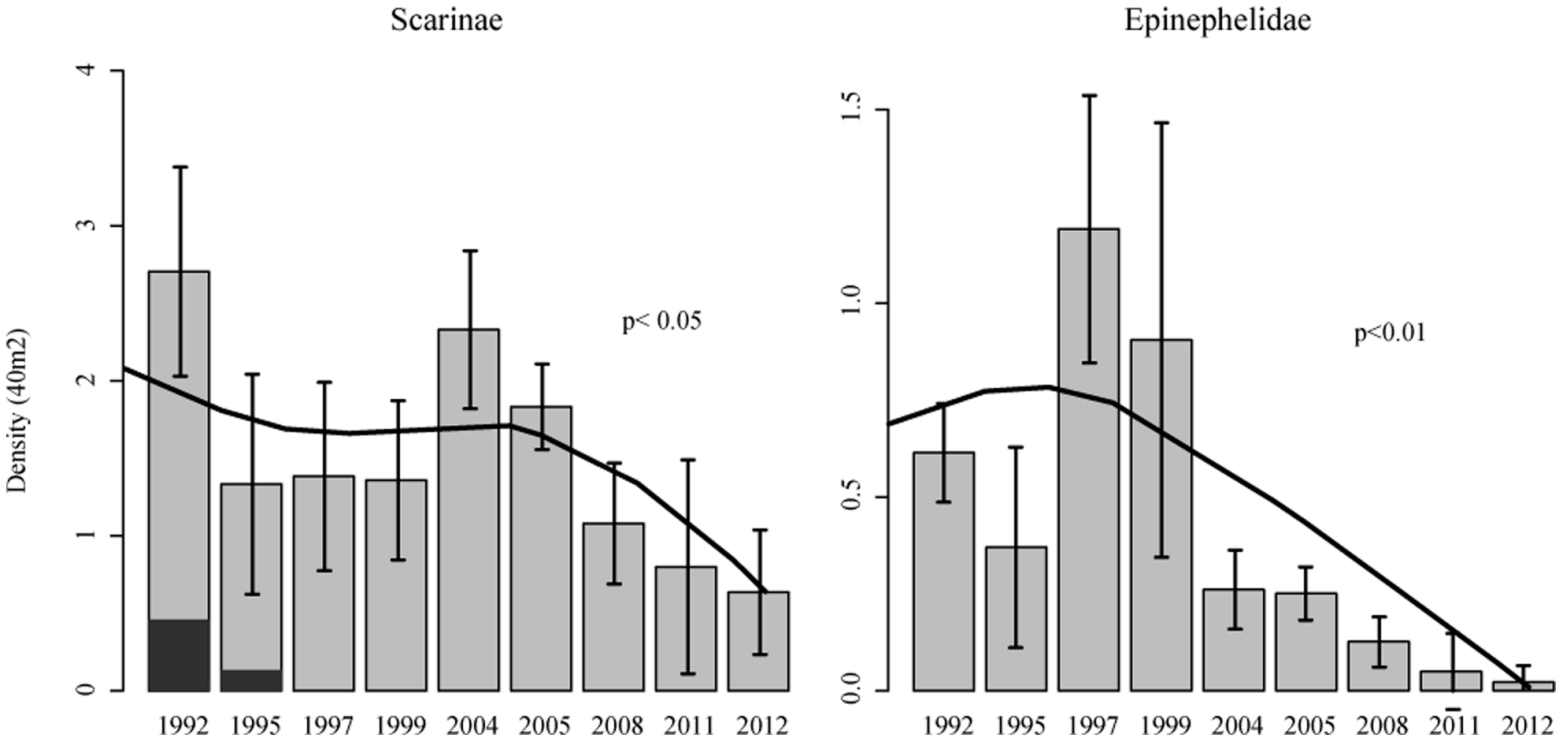
Mean densities per 40 m^2^ of Scarinae and Epinephelidae species in Arraial do Cabo from underwater visual census. In the Scarinae plot, black portion of bars correspond to the contribution of *Scarus trispinosus* to densities.

Data from visual census campaigns conducted in the region from 1992 to 2012 – revealed a declining trend in the local densities of *Scarus trispinosus*, in fact, individuals of the species are not recorded on transects conducted in the region since 1995 (Fig. 4). The decline in *S. trispinosus* is followed by an overall decline of Scarinae (Labridae) species. For Epinephelidae species, the situation is similar, with decreasing densities in local reef fish communities from 1997 onwards (Fig. 4).

### Shifting baselines across multigear fisheries

Out of 214 interviewed fishermen, ∼83% (n = 177) recognized reductions in the abundance of exploited fish species in the region of Arraial do Cabo. Less experienced fishermen are less aware of overexploitation, indicating the occurrence of the SBS among local fishermen. Significant age-related differences were evident in assessed fishing communities in terms of respondent perception regarding fisheries declines. Experienced line, beach seine and spearfishermen mentioned a greater number of overexploited species when compared to less experienced ones (Table 1).

**Table 1:** Mean number of overexploited fish species, new target species and depleted sites mentioned by fishermen from different fishing gears and according to experience categories.

Fishing modality	Fishers experience category (n)	Overexploited species (mean ±SD)	ANOVA*	New target species (mean ±SD)	ANOVA*	Depleted sites (mean ±SD)	ANOVA*
Line	Beginners (22)	2.0±1.0	*f* = 139.3	1.6±1.1	*f* = 35.7	2.0±1.0	*f* = 135
	Intermediate (25)	5.5±1.8	*p*<0.001	3.1±1.5	*p*<0.001	4.5±1.9	*p*<0.001
	Experienced (21)	10.5±1.7		6.0±1.0		11.3±2.3	
Beach seine	Beginners (23)	2.3±1.5	*f* = 76.1	1.4±1.5	*f* = 60.8	2.4±2.1	*f* = 63.6
	Intermediate (26)	6.0±2.2	*p*<0.001	3.4±1.1	*p*<0.001	5.5±2.0	*p*<0.001
	Experienced (21)	9.0±1.5		5.5 ± 1.9		10.5±3.1	
Spearfishing	Beginners (25)	1.4±0.9	*f* = 187.7	1.8±0.7	*f* = 107.2	1.8±1.4	*f* = 133.5
	Intermediate (28)	5.8±1.5	*p*<0.001	5.1±1.7	*p*<0.001	6.3±1.7	*p*<0.001
	Experienced (23)	8.9±1.7		7.4±1.9		11.4±2.9	

*f and *p*-values of each ANOVA (one-way) test.

All values associated to different age categories were significantly different according to ANOVA (one-way) and Student-Newman-Keuls comparison test (p<0.05).

We detected significant experience-related differences in terms of respondent perception of shifts in target species (Table 1). Not only for line and beach seine fishermen (line fishing: experienced  = 6.1, intermediate  = 3.1 and beginners  = 1.6; beach seine: experienced  = 5.5, intermediate  = 3.4 and beginners  = 1.4), but also for spearfishing there were differences between the mean number of species cited by each category (spearfishing: experienced  = 7.4, intermediate  = 5.1 and beginners  = 1.8) (see Table 1). For instance, surgeonfishes (e.g. *Acanthurus* spp.) were identified as targets exclusively by beginner spearfishermen. The mean number of sites mentioned as depleted by different fishermen categories also suggests the occurrence of the SBS. The more experienced and older fishermen referred to a greater number of overexploited sites when compared to younger, less experienced fishermen. For instance, experienced line fishermen cited approximately 11 sites, while intermediate and beginners referred to 4 and 2 sites, respectively (see Table 1).

## Discussion

### From local to global: the impacts of changes in fishermen's perceptions

Since Daniel Pauly reported the global phenomenon that he termed ‘the shifting baseline syndrome’ (SBS) [27], much supporting evidence have been reported across marine [29], [53], freshwater [41], [54] and terrestrial ecosystems [43], [55]. Fishermen gather their environmental references based on personal experiences, which may lead to perception differences among generations. In Arraial do Cabo, experienced line and beach seine fishermen often mentioned the old days when top predators such as the sharks *Carcharhinus milberti*, *Squatina argentina*, *Isurus oxyrinchus* and the shovelnose ray *Rhinobatos percellens* were abundant in their catches, being witnesses to the decline of those species. Even though most of the older beach seine fishermen (73%) recognized the shark *Carcharhinus maculipinnis* as overexploited, beginners believe that the species is naturally rare in the region. Also, contrary to older fishermen, young spearfishermen did not mention the snappers *Lutjanus analis, Lutjanus cyanopterus*, and the groupers *Mycteroperca microlepis* and *Epinephelus morio* as depleted. The decline of these fish species forced fishermen to change their target species. Those species that were not important to fisheries in the past – or were only utilized as bait – turned out to be today's main targets. This replacement process is occurring slowly and gradually over the years, making it more difficult for future generations of fishermen to perceive these changes. Experienced and older fishermen have seen the effects of overexploitation upon marine ecosystems for many more years, whereas young fishermen begun fishing when stocks were either reduced or on the path for depletion. This led young fisherman to assume that sharks and large groupers were naturally rare in Arraial do Cabo, and that overexploited fishing grounds were still very productive. Thus perceptions are subjected to the effects of time and possibly compromised knowledge transmission between community members [30].

Baseline shifts regarding the past conditions of the world's oceans might lead to a collective amnesia, making people more tolerant to population declines and biodiversity losses [56]. Despite the fact that fishermen are usually in contact with nature on a daily-basis, exhibiting greater awareness of environmental changes, baselines shift rapidly even among them [29], [41]. In Arraial do Cabo, sixty years of fisheries exploitation and impacts upon marine ecosystems have affected the environmental baselines of three consecutive generations. Such changes in the perceptions of fishermen regarding the status of marine ecosystems are of special concern given the importance of LEK to adjust and improve our understanding of the oceans' past conditions.

A possible short-term solution to slow the reference loss process is to recreate old conditions of marine life through historical data [4] such as films, photos [57], booklets and reports [31], [50] of early explorers. Such kind of data can provide important detail on former species size and catch composition [30], potentially improving our knowledge on the effects of overfishing. Recreating past environmental baselines is a unique opportunity to assess the historical changes that ecosystems have undergone, as well as for improving social awareness on the status of biodiversity. Recent studies have pointed that underestimating empirical data is a mistake [27], [29], [45], [56], [58], [59], especially in places like Arraial do Cabo, where written records on species' former size and abundance are rare.

When assessing fishing impacts, few studies make such parallel use of LEK and species population status, mostly because LEK is considered anecdotal, non-scientifically sampled, and of limited application among scientists and managers [40], [60]. Our work is among the first to combine landing, underwater visual census and LEK data to assess the temporal patterns of exploited fish populations [58], [61]. Combining data from different sources renders more robust and reliable assessments, which are especially important in times where decision makers require scientific certainty. More importantly, local management and conservation efforts will certainly gain more commitment from fishermen when their own experience is taken into account on the establishment of management strategies.

### Fishing collapse in the Southwestern Atlantic

The combined methodologies utilized to assess multigear fisheries reveal the decline of different fish species in the region of Arraial do Cabo. The decline of the bluefish (*P. saltatrix*) fishery revealed through information provided by fishermen was supported by evidence from landing data. The declining trend for Epinephelidae species (*E. marginatus, Mycteroperca bonaci*, *M. acutirostris* and *M. microlepis*) in the subtropical reefs of Arraial do Cabo has been demonstrated through information provided by fishermen, and through data from underwater visual census campaigns conducted in the region. Another study for the region underscores recent declines in the abundance of groupers using the underwater visual census technique [17]. Not only the abundance of fish is diminishing, but also the larger individuals are consistently more difficult to capture and sight in the field [17]. Overfishing is affecting the size of fish individuals, removing the largest ones at an unsustainable rate, and preventing the smallest ones from reaching large sizes. This can seriously compromise fish population rebound since larger and older females produce greater quantity and better quality offspring when compared to younger fish [62]. Most of these grouper species are important fishing targets elsewhere in the Brazilian coast, and the overfished state of populations has been demonstrated by different studies [17], [20]. Moreover, they are highly vulnerable to overexploitation given its critical combination of biological attributes [25].

As populations of top predators collapse, other large-bodied species at lower trophic level become new targets, as is the case of the endemic greenbeak parrotfish, *Scarus trispinosus*. Being the largest of all Brazilian parrotfishes, the greenbeak was the first herbivorous-detritivorous reef fish targeted by spearfishermen, but other large-bodied parrotfishes of the genus *Sparisoma* also became targeted in the last few years. Different species of parrotfishes perform specific functional roles, some reported as critical in maintaining the resistance and resilience of reef ecosystems [63], [64]. Along the Brazilian coast, populations of *S. trispinosus* have strongly declined due to spearfishing [17], and the species is considered ecologically extinct in Arraial do Cabo [50]. The species was recently listed by the IUCN [65] as Endangered (EN) yet there is no regulation or national listing to guarantee its protection [25].

Here we have shown the decline of reef fisheries in the Southwestern Atlantic from multiple sources of information: local ecological knowledge from multigear fisheries (line, beach seine and spearfishing), fisheries landing data and even underwater visual census data. The pattern identified for six different target species follows a similar pattern of global deterioration of marine ecosystems reported elsewhere [1], [66]. In the Southwestern Atlantic, as elsewhere in the world, fishing has moved from upper to lower trophic levels: groupers used to be fisheries' major target, but today, parrotfishes and even surgeonfishes are targeted. If going back to the early days of the 1960's tell us a very different story on fish abundance and size in the region of Arraial do Cabo, one could only imagine what a 150 or 200-year-old baseline would inform us on those fish species. We emphasize the importance of combining LEK with scientific data in order to improve our understanding of the status of marine habitats and its associated biodiversity, but the occurrence of changes in environmental baselines must be taken into account. Only through a clear picture of the status of natural resources in the past can we help people improve their awareness of the importance of preserving natural resources and ecosystem services.

## Supporting Information

File S1 **File contains Questionnaire and Figure S1.** Questionnaire applied in interviews with fishermen in Arraial do Cabo. Figure S1. Catch trends for *Sparisoma* (Scarinae) species reported by spearfishermen in Arraial do Cabo. Linear regressions of the largest individual caught (kg) and best day's catch (number of individuals) of the parrotfishes *Sparisoma axillare, S. amplum* and *S. frondosum* that spearfishers remembered landing (from top to bottom).
(DOCX)

Data S1 **Landing data registered in Arraial do Cabo for the bluefish** ***Pomatomus saltatrix*** **, and fish abundances (groupers and parrotfishes) recorded in underwater visual census campaigns, also in Arraial do Cabo region.**
(XLSX)
