# Supplementary material for: Local Ecological Knowledge and Scientific Data Reveal Overexploitation by Multigear Artisanal Fisheries in the Southwestern Atlantic
Source: PLoS One. 2014 Oct 15;9(10):e110332. doi: 10.1371/journal.pone.0110332 (PMC4198246; doi:10.1371/journal.pone.0110332)
Supplement: File S1 — File contains Questionnaire and Figure S1. Questionnaire applied in interviews with fishermen in Arraial do Cabo. Figure S1. Catch trends for Sparisoma (Scarinae) species reported by spearfishermen in Arraial do Cabo. Linear regressions of the largest individual caught (kg) and best day's catch (number of individuals) of the parrotfishes Sparisoma axillare, S. amplum and S. frondosum that spearfishers remembered landing (from top to bottom). (DOCX) [file pone.0110332.s001.docx]

**File S1. Questionnaire applied in interviews with fishermen in Arraial do Cabo.**

This questionnaire was randomly applied to fishers encountered at the beach. All interviews were conducted in private in order to minimize the effects of other fishers’ presence on the answers given. The older, most experienced fishers who were retired from fishing activities were visited and interviewed in their households. We obtained verbal consent from participants before conducting interviews, and fishers were informed on the purpose of the research.

***Line and beach seine fishing modalities***

Name: _________________________________________________________

Age: _______

1. How long have you been fishing? __________

( ) Beginner (< 15 years)

( ) Intermediate (16-30 years)

( ) Experient (>30 years)

2. Is fishing your only source of income? ( ) Yes ( ) No

3. How would you describe the current conditions of fish stocks regarding abundance?

( ) unaltered ( ) declined ( ) augmented

4. Do you know any overexploited species by fisheries in this region? ( ) Yes ( ) No

If Yes, which species? _________________________________________________________

5. Do you know any species that was formerly discarded or only used as bait that today is a fishing target? ( ) Yes ( ) No

If Yes, which species? _________________________________________________________

6. Do you know any place in this region that was considered a good fishing site due to the abundance and productivity of fish, but nowadays is considered overexploited? ( ) Yes ( ) No

7. If you were to point one area to be protected from fishing, which area would that be? Why?

8. What (management) measures do you consider important to improve the fisheries’ productivity in this region?

9. Bluefish (*Pomatomus saltatrix*) catches

What was the largest amount (kg) of fish of this species that you captured? ________ kg

When? __________

***Spearfishing modality***

Name: _________________________________________________________

Age: _______

1. How long have you been fishing? __________

( ) Beginner (< 8 years)

( ) Intermediate (8-15 years)

( ) Experient (>15 years)

2. Is fishing your only source of income? ( ) Yes ( ) No

3. What is the purpose of spearfishing for you? ( ) Leisure ( ) Consumption ( ) Commercialization

4. Sperfishing modality: ( ) Free diving ( ) Scuba diving ( ) Compressor diving

5. How would you describe the current conditions of fish stocks regarding abundance?

( ) unaltered ( ) declined ( ) augmented

6. Do you know any overexploited species by fisheries in this region? ( ) Yes ( ) No

If Yes, which species? _________________________________________________________

7. Do you know any species that formerly was not captured by spearfishers, but became a target of spearfishing? ( ) Yes ( ) No

If Yes, which species? _________________________________________________________

8. Do you know any place in this region that was considered a good fishing site due to the abundance and productivity of fish, but nowadays is considered overexploited? ( ) Yes ( ) No

7. If you were to point one area to be protected from fishing, which area would that be? Why?

8. What (management) measures do you consider important to improve the fisheries’ productivity in this region?

9. Fish catches (all groupers and parrotfishes)

What was the largest amount of fish of this species that you captured (n individuals)? ________

When? __________

What size is the largest individual (of this species) you ever caught? ________ kg

When was this large individual caught? __________


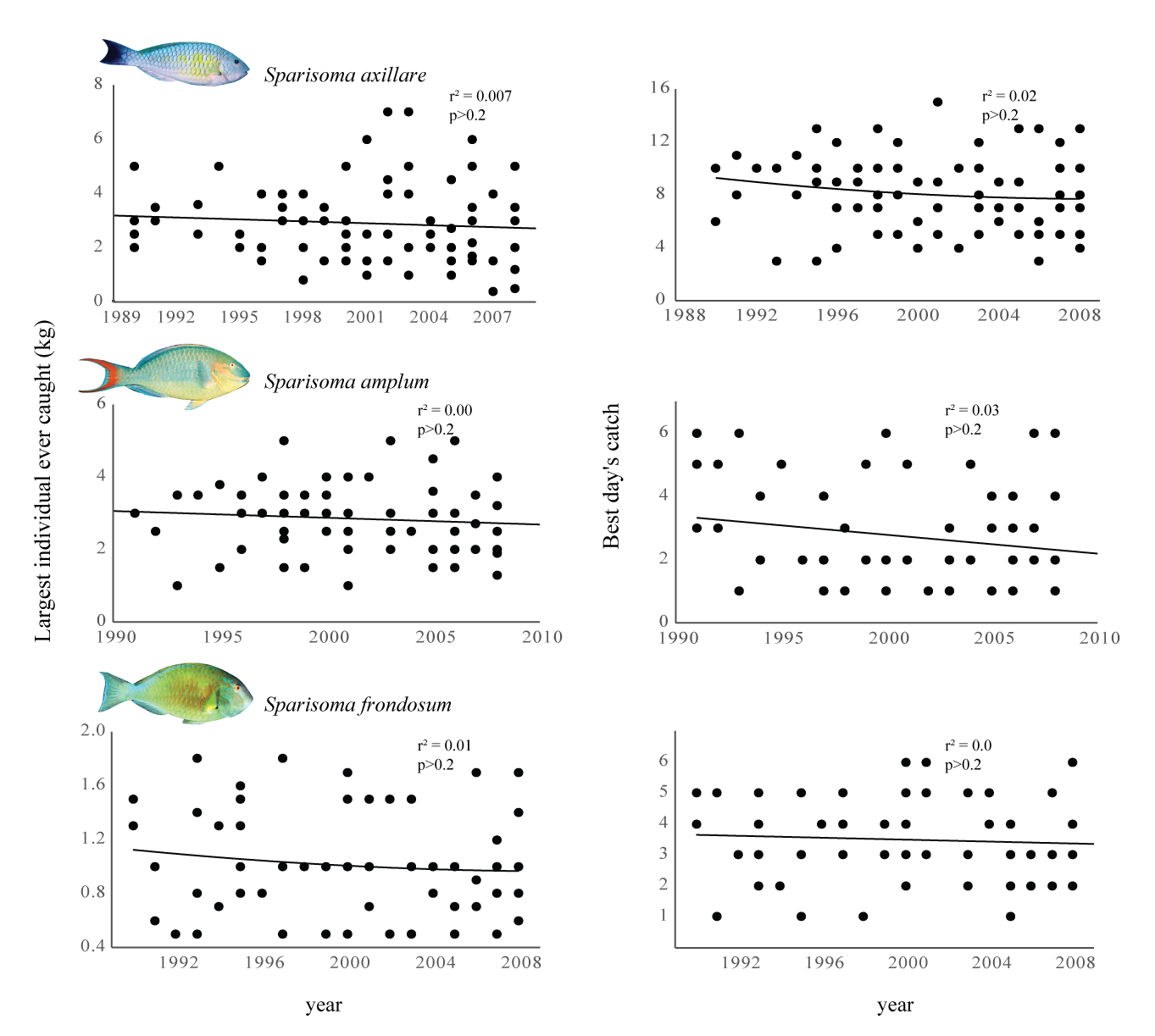


**Figure S1**. **Catch trends for *Sparisoma* (Scarinae) species reported by spearfishermen in Arraial do Cabo.** Linear regressions of the largest individual caught (kg) and best day’s catch (number of individuals) of the parrotfishes *Sparisoma axillare, S. amplum* and *S. frondosum* that spearfishers remembered landing (from top to bottom).
